# Supplementary material for: Secondary metabolites of the invasive tree, Callery pear (Pyrus calleryana), provide support for the empty niche theory of invasion
Source: PLoS One. 2025 Jul 24;20(7):e0327841. doi: 10.1371/journal.pone.0327841 (PMC12289043; doi:10.1371/journal.pone.0327841)
Supplement: Attachment — Submitted filename: Response to Reviewers.docx [file pone.0327841.s003.docx]

Reviewer #1: Secondary metabolites of the invasive tree, Callery pear (Pyrus calleryana), provide support for the empty niche theory of invasion

General comments:
I agree that the results only confirms the ‘empty niche theory’, but only partially. Of course there is no congener species in the US, suggesting existence of empty niche, but I think the other aspects of species biology are also important for invasion: the scleromorphic leaves (thick and glossy, related to drought resistance), quick growth (up to 90 cm per year), high generative and clonal growth potential – root suckering, and seed dispersal by animals. The unique ‘battery’ of secondary metabollites seems help the plant additionally get an advantage in resistance to abiotic and biotic stresses, after establishment.

**Thank you for your helpful comments. I did add L265-266 and 268-270 to the discussion to revisit some of the biological reasons that CP is so successful.**

Methods:
- please provide more information about study areas (geographical location, maps, maybe and environmental factors that can potentially affected the metabolite profiles

**Thank you for the suggestion. I added a map (Figure 1) with all sites represented. I also added a table with the number of each species found at each site, since they were not evenly distributed. I have also added Lines 101-110 with environmental and climate information about the sites. L234-236 in the discussion addresses the limitations of collecting in the same types of sites and call for expanded collections across different site types.**

- statistical analyses – please provide the literarure and software used.

**Thank you! This was a total oversight on my part. I added this information to L161 and also added Pang et al. 2022 as the citation.**

Detailed comments:
Line 94 I suggested to state the hypotheses research questions directly to link the comparison of secondary metabolities

**Added L94-96: “…** **and predicted that the profile of CP would be significantly different from that of both peach and black cherry.”**

Line 99 …”S.C”. – please use full geographic names and geographical location (s) of the study area for clarity

**Changed to South Carolina**

Reviewer #2:

 The study provides a comparative analysis of secondary metabolite profiles between invasive (Pyrus calleryana) and native/non-invasive plants (Prunus serotina and P. persica), offering preliminary insights into potential biochemical mechanisms underlying plant invasion. The focus on flavonoids and their ecological implications aligns with contemporary research on allelopathy and niche theory. However, the current data are insufficient to robustly support the conclusions and most of the discussion are speculative. My key concerns are as follows:

Lack of direct evidence: The study identifies metabolite differences but does not demonstrate their functional roles (e.g., allelopathy, microbial interactions).

Speculative discussions: Claims about allelopathic effects or soil microbial impacts are unsupported by experimental data.

Narrow scope: Sampling was limited to leaf tissue in one season, omitting root exudates, rhizosphere microbes, and temporal variation.

To substantiate the conclusions, the authors should conduct complementary experiments:
i) Microbial community analysis: Compare rhizosphere soil microbiomes between P. calleryana and native plants to test for flavonoid-driven shifts.

ii) Common garden bioassays: Apply flavonoids to native plants to quantify growth inhibition or microbial community changes.

iii) Competition trials: Co-culture P. calleryana with native species under controlled conditions to assess competitive outcomes linked to metabolite production.

iv) Expand metabolite profiling: Include root tissues and seasonal sampling to evaluate spatial/temporal dynamics of flavonoid production.

**Thank you for the thoughtful comments. I fully agree that, without these additional experiments, the argument for allelopathy is tenuous. In my original discussion I tried to steer clear of specifically suggesting allelopathy in CP and give the study limitations which prevent me from making those claims. The empty niche theory of invasion does not equate directly to allelopathy, so I believe my original claims of evidence in favor of this theory still hold water. Unfortunately, I do not have the capacity to conduct these experiments as this was a side quest for a project which has since ended. However, there are currently projects underway including greenhouse controlled experiments to look at establishment and competition among CP and other species. In the updated discussion, I reference the studies outlined by R2 as necessary future research to confirm allelopathy in CP.**

**L234-236: In my original discussion I address the limitations of a single season of sampling and suggest that future research do additional sampling to look at seasonal changes to the SM profile.**

**Discussion additions:**

**L201-204: added next steps of quantification of SM**

**L237-241: added future research to apply flavonoids to natives and non-native non-invasive plants to look at both the effects on plant growth and defenses as well as the effects on rhizosphere communities**

**L252-255: added future research needed to look at SM of different tissue types**

**L266: added “and tissue types”**

When submitting your revision, we need you to address these additional requirements.

1. Please ensure that your manuscript meets PLOS ONE's style requirements, including those for file naming. The PLOS ONE style templates can be found at

**Updated Supp. Table info in paragraph to match style.**

**I found that all info is actually in Supp Table 1 so I removed the reference to Supp Table 2 and updated that to Supp Table 1. I also made sure to match the reference for the table to the guidelines.**

**Updated References heading to match style.**

**Updated Statistical analyses heading to match style.**

**Added “Supplemental information” section with appropriate table title and description. I made sure this matches the style guidelines as well.**

2. In your Methods section, please provide additional information regarding the permits you obtained for the work. Please ensure you have included the full name of the authority that approved the field site access and, if no permits were required, a brief statement explaining why.

**Added L111-112 regarding permits for collections.**

3. Please upload a copy of Supporting Information Table 1 & 2 which you refer to in your text on page 6.

**See above comment re: supplemental tables.**
